# Specific ophtalmologic changes in late onset familial amyloid polyneuropathy (FAP) portuguese patients

**DOI:** 10.1186/1750-1172-10-S1-P63

**Published:** 2015-11-02

**Authors:** Natalia Ferreira, David Dias, Teresa Coelho

**Affiliations:** 1 Ophthalmology Department - Centro Hospitalar do Porto – Hospital Santo António (HSA), Porto, Portugal; 2 Neurology Department – Centro Hospitalar do Porto – HSA - Unidade Corino de Andrade, Porto, Portugal

## Purpose

Report ocular manifestations in late onset familial amyloid polyneuropathy (FAP) patients.

## Methods

Retrospective observational consecutive case series of 20 late onset FAP patients. Demographic data, TTR mutation involved, age at beginning of disease, period of evolution of disease, liver transplant or medical treatment, ophthalmological alterations and previous ocular surgeries were evaluated.

## Results

Thirteen patients were female. The mean onset age was 58 years and average evolution time of the disease was 5, 6 years. All patients were TTR Met30 and 2 patients were compound heterozygous TTR met30 met119. Four patients had been submitted to liver transplant and nine were on Tafamidis treatment. Amyloid deposits on anterior lens surface were observed in 15 eyes (37,5%), scalloped pupil in 8 eyes (20%) and vitreous opacities in 23 eyes (57,5%). Nine had underwent vitrectomy. Glaucoma was present in 13 eyes and 4 have been submitted to surgery.

## Conclusion

Ocular manifestations are common in late onset FAP patients. Vitreous opacities were the most frequent specific alteration. Ophthalmologist has an important role in follow-up of FAP patients to accurately treat sight-threatening manifestations.

